# Marine compounds inhibit growth of multiple myeloma *in vitro* and *in vivo*

**DOI:** 10.18632/oncotarget.3362

**Published:** 2015-01-31

**Authors:** Normann Steiner, Domenico Ribatti, Wolfgang Willenbacher, Karin Jöhrer, Johann Kern, Christian Marinaccio, Miguel Aracil, Luis F. García-Fernández, Guenther Gastl, Gerold Untergasser, Eberhard Gunsilius

**Affiliations:** ^1^ Laboratory for Tumor Biology & Angiogenesis, Innsbruck Medical University, Innsbruck, Austria; ^2^ Department of Internal Medicine V, Innsbruck Medical University, Innsbruck, Austria; ^3^ Tyrolean Cancer Research Institute, Innsbruck, Austria; ^4^ Department of Basic Medical Sciences, Neurosciences, and Sensory Organs, University of Bari Medical School, Bari, Italy; ^5^ National Cancer Institute “Giovanni Paolo II”, Bari, Italy; ^6^ PharmaMar R&D, Colmenar Viejo, Madrid, Spain; ^7^ Oncotyrol GmbH, Innsbruck, Austria

**Keywords:** Marine drugs, Angiogenesis, Multiple Myeloma, CAM, Xenografts

## Abstract

**Purpose:**

The prognosis of patients with multiple myeloma (MM) is still dismal despite recent improvements achieved by introducing new therapeutic agents. However, there remains an urgent need for progress in myeloma drug development. We here show that novel marine-derived compounds can exert potent anti-myeloma activity.

**Experimental Design:**

Nine marine-derived compounds were applied at low nM concentrations (0.1-100 nM) to MM cell lines (OPM-2, NCI-H929, U266, RPMI-8226), to primary human myeloma cells and to peripheral blood mononuclear cells. Apoptosis was determined by flow cytometry. In addition, eGFP-transgenic MM cell lines growing with mesenchymal cells from bone marrow were used to visualize tumors by fluorescence stereomicroscopy. Anti-myelomaactivities were studied *in vitro* in 3D spheroids and *in vivo* in myeloma xenografts on chicken embryos. Tumor size was analyzed by measuring GFP content with a GFP ELISA. Anti-angiogenic activities of compounds were tested in an *in vivo* gelatin sponge assay with conditioned media from primary bone marrow-derived endothelial cells.

**Results:**

We identified a subset of marine compounds with strong anti-myeloma activity *in vitro* and *in vivo.* Moreover, some of the compounds inhibited myeloma-related angiogenesis in the *in vivo* gelatin sponge assay. They merit further drug development to improve treatment options for MM.

## INTRODUCTION

Multiple myeloma (MM) is the second most frequent hematological malignancy [1], a malignant B-cell neoplasm with rising prevalence that causes considerable morbidity, mortality and health care expenditures [2, 3]. Although the last decade has seen considerable improvement in overall and progression-free survival due to the introduction of new drugs as proteasome inhibitors like bortezomib and carfilzomib and immunomodulatory agents including thalidomide, lenalidomide and pomalidomide [4, 5], in most cases MM remains an incurable disease with inevitable disease progression due to clonal evolution, development of drug resistance and fatal outcome [4].

MM is a highly heterogeneous malignancy characterized by complex primary and secondary cytogenetic and molecular aberrations with varying clinicopathological features and disease course [6, 7]. Due to intraclonal and interclonal tidings MM cells constitute a “moving therapeutic target” [8]. Therefore, new drugs might supplement the current therapeutic armamentarium by targeting putative driver genes and signaling pathways in MM cells and affecting the myeloma stromal niche in the bone marrow.

In this regard, the marine environment with its large biodiversity constitutes a rich source of potential therapeutic compounds. Systematic high-throughput screening has identified the antitumor potential of a variety of marine-derived compounds with significant biological activities *in vitro* and *in vivo*. Several marine lead compounds are currently under development as cancer therapeutics. Plitidepsin (Aplidin), an anticancer agent originally isolated from the tunicate Aplidium albicans and currently obtained by total synthesis, is being tested in a phase III trial in patients with relapsed or refractory multiple myeloma (registered with www.clinicaltrials.gov, number NCT01102426). At the molecular level, Plitidepsin is a potent inducer of apoptosis. This effect has been related to its interaction with eEF1A and the generation of early oxidative stress, which triggers the sustained activation of JNK and p38 MAPKs [9] and leads to endoplasmic reticulum (ER) stress and subsequent unfolded protein response (UPR) activation (Drug Discovery Area, PharmaMar S.A.U., unpublished data).

In this study, we assayed a selected set of marine substances with respect to anti-myeloma activities *in vitro* and *in vivo* against standard anti-myeloma compounds, such as bortezomib, and substances in advanced development like Plitidepsin as reference.

## RESULTS

### Screening for compounds with anti-myeloma activity using myeloma cell lines *in vitro*

Marine compounds were tested for their ability to induce apoptosis in human MM cell lines. For this purpose, cell viability was measured by flow cytometry 24h after incubation with increasing concentrations of test compounds. Most compounds showed anti-myeloma activity *in vitro* when tested at concentrations of 100 nM in commercially available human MM cell lines growing under standard culture conditions (Table 1, Supplementary Figure 1). Plitidepsin, Zalypsis, PM00113, PM01215, PM02781 and Thiocoraline A induced significant apoptosis in all MM cell lines at 100 nM. A strongly dose-dependent effect was seen with Zalypsis, PM00113 and Thiocoraline A; these compounds were even effective at a concentration of 10 nM in one or more cell lines inducing apoptosis like the reference compound bortezomib. Notably, at 100 nM concentration compounds Plitidepsin, Zalypsis, PM00113 and Thiocoraline A also induced apoptosis of peripheral blood mononuclear cells (PBMNC) from five healthy donors (Supplementary Figure 2A) comparable to bortezomib 100 nM (apoptotic rate increased from 11.3 ± 3.2 in control cells to 28.0 ± 4.3% in bortezomib-treated cells, data not shown).

**Table 1:** Induction of apoptosis by marine-derived compounds in MM cell lines (% of living cells)

Cell type	Concentration	Con.	Bortezomib	Plitidepsin	Lamellarin D	Elisidepsin	ES-285	Zalypsis	PM00113	PM01215	PM02781	Thiocoraline A
**OPM-2**	10 nM	80	49*	78	73	77	76	44*	39*	77	74	76
	100 nM		44*	52*	53*	78	78	42*	39*	62*	39*	62*
**U266**	10 nM	80	22*	75	77	76	76	40*	42*	71	71	70
	100 nM		20*	52*	71	76	76	38*	39*	59*	54*	62*
**NCI-H929**	10 nM	73	6*	67	70	69	73	52*	41*	69	66	55*
	100 nM		5*	6*	36*	68	72	7*	7*	10*	9*	9*

**MM cell lines (OPM-2, U266, NCI-H929) were incubated with increasing concentrations of drugs for 24h and then analyzed by flow cytometry.** Annexin-V^neg^ and 7AAD^neg^ cells were defined as viable. Data were calculated from triplicate results for each drug; 10 nM or 100 nM. Highlighted numbers indicate significant killing of myeloma cell lines. Stars indicate p values <0.05. Con.: control.

### Screening for compounds with anti-myeloma activity using bone marrow aspirates of patients with multiple myeloma

Anti-myeloma activity of the marine compounds was examined *in vitro* in ten samples of primary human multiple myeloma cells. Clinical data of all patients are provided in Supplementary Table 1. Whole bone marrow, containing MM cells, normal hematopoietic and stromal cells of the microenvironment, was cultivated *in vitro* and incubated with increasing concentrations of target compounds. After 24h primary MM cells were identified by expression of CD45^low^ and CD38^high^ in flow cytometry with gating for viable myeloma cells (annexin-V^neg^/7AAD^neg^). In contrast to commercially available MM cell lines, primary patient MM cells cultivated in context with the respective cells of their microenvironment were remarkably more sensitive to applied drugs (Table 2, Supplementary Figure 3). In fact, most of the drugs effectively induced apoptosis even at a concentration of 10 nM. In detail, Plitidepsin, Zalypsis, PM00113 and Thiocoraline A applied at a concentration of 10 nM showed significant anti-myeloma activity against primary MM cells *in vitr*o. PM01215 and PM02781 at 10 nM demonstrated apoptotic activity only in individual patients (Table 2) and thus were excluded from further analysis. MM cells from one patient (MM#3, clinically refractory to bortezomib, see Table 2) were not sensitive to bortezomib at all, but were readily killed *in vitro* with marine compounds. MM cells from patient MM#1 (newly diagnosed MM), MM#5 (progressive disease with plasma cell leukemia), MM#6 (progressive disease), MM#9 (progressive disease) and MM#10 (progressive disease), all five considered high-risk patients, showed a significant apoptosis response when treated with two to four of the marine-derived compounds. All but one patient sample (MM#7; high-risk myeloma, progressive disease) were sensitive to marine-derived compounds. With regard to peripheral blood mononuclear cells (PBMNC, n=5) all tested compounds displayed no significant apoptotic effects at concentrations of 10 nM, as determined by staining for annexin-V^neg^ /7AAD^neg^ and flow cytometric analysis (Table 2, Supplementary Figure 2A).

**Table 2:** Induction of apoptosis by marine-derived compounds in primary myeloma cells and PBMNC (% of living cells)

Patient	Con.	Bortezomib	Plitidepsin	Lamellarin D	Elisidepsin	**ES-285**	Zalypsis	**PM00113**	**PM01215**	**PM02781**	Thiocoraline A
**MM#1**	79	12*	73	81	77	82	15*	18*	77	83	65*
**MM#2**	79	59*	75	72	74	82	62*	67*	66*	73	62*
**MM#3**	82	77	51*	83	85	81	16*	15*	70	70	76
**MM#4**	67	6*	46*	63	65	53*	10*	9*	61	58	58
**MM#5**	89	20*	74	86	88	86	5*	5*	76	69*	82
**MM#6**	80	12*	26*	72	73	79	5*	6*	51*	54*	69*
**MM#7**	82	50*	80	82	82	82	83	80	79	78	74
**MM#8**	72	29*	18*	74	70	71	16*	14*	64	72	58*
**MM#9**	66	5*	64	68	67	64	18*	10*	65	60	60
**MM#10**	74	27*	39*	74	65	72	18*	24*	65	70	56*
**PBMNC**	81	76	83	85	84	82	82	82	81	82	80

**Exemplary experimental data from multiple myeloma patients (n=10).** MM cells were defined as CD38^high^ and CD45^low^. Peripheral blood mononuclear cells (PBMNC) from donors served as control for toxicity in primary cells. Data from triplicate results of 10 nM concentrations of each drug (S1-S9). Mean of triplicate results, stars indicate p values <0.05. Con.: control. Highlighted compounds efficiently killed primary MM cells at concentrations of 10 nM, but not primary PBMNC in at least five patient samples. These compounds were used for further studies in MM *in vitro* and *in vivo* models.

Based on these findings made in primary MM cell cultures, the four compounds Plitidepsin, Zalypsis, PM00113 and Thiocoraline A, effective at 10 nM, were used for further analysis in 3D *in vitro* and *in vivo* assays of MM cells with the respective mesenchymal microenvironment.

### *In vitro* analysis of selected marine compounds in 3D *in vitro* multiple myeloma spheroid assays

**Figure 1:**
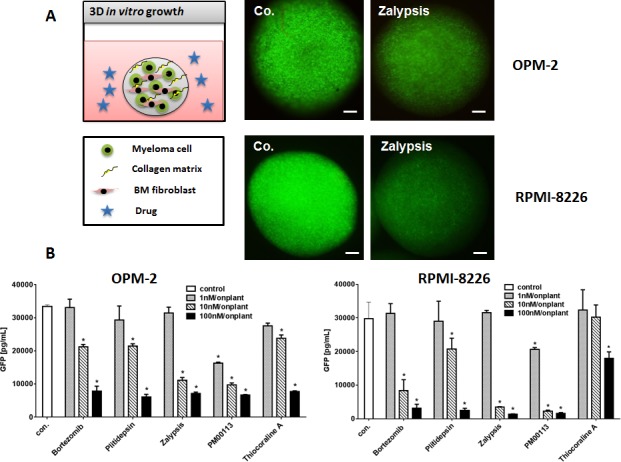
3D Multiple Myeloma Model (A) Human multiple myeloma cell lines were lentivirally transfected and selected to stably express eGFP. OPM-2^eGFP^ and RPMI-8226^eGFP^ cells were cultivated in hanging drops with primary human bone marrow mesenchymal cells and collagen type I as extracellular matrix component. Spheroids were incubated with culture medium (co.), and respective concentrations (1-100 nM) of all target compounds effective on primary MM cells. MM cell lines were grown for 72h in spheroids and photographed daily using a stereo-fluorescence microscope. Bars indicate 500 μm. (B) Single spheroids were homogenized in lysis buffer and subsequently measured in a GFP ELISA. GFP concentrations of single spheroids were calculated (n=6, Mean ± SEM). Stars indicate p values <0.05. Con.: control.

Due to the limitations of culturing primary human MM cells *in vitro* we established a 3D *in vitro* culture model of human MM cell lines using a collagen extracellular growth matrix and supportive primary human mesenchymal cells from bone marrow. MM cell lines transgenic for eGFP allow visualization and quantification of MM tumor mass after 3D growth in spheroids (Figure 1A). Both MM cell lines OPM-2^eGFP^ and RPMI-8226^eGFP^ were cultivated for three days in the presence of increasing concentrations (1-100 nM) of the respective target compounds, and tumors were visualized by GFP expression (Figure 1A). All target compounds significantly inhibited MM growth at concentrations of 10 and 100 nM in OPM-2^eGFP^ and RPMI-8226^eGFP^ spheroids.

Tumor cell mass was quantified after spheroids were homogenized and eGFP content was measured with a GFP-ELISA (Figure 1B). With regard to the applied drug molarity, Zalypsis and PM00113 were the most potent inhibitors in OPM-2^eGFP^ and RPMI-8226^eGFP^ 3D spheroids, reaching or even exceeding the anti-myeloma activity of bortezomib. Noteworthy, in MM 3D spheroids none of the marine drugs induced apoptosis in mesenchymal cells, even at concentrations of 100 nM (data not shown). These data confirm our analysis of apoptosis induction in standard culture of primary human mesenchymal cells derived from bone marrow and exposed to marine compounds, which cells were significantly more resistant to apoptosis than were MM cells (Supplementary Figure 2B).

### *In vivo* analysis of selected marine compounds using myeloma xenografts in chicken embryos

**Figure 2:**
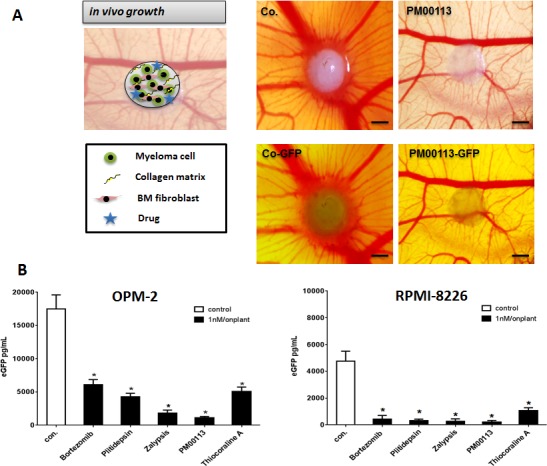
Multiple Myeloma Xenograft Model (A) OPM-2^eGFP^ and RPMI-8226^eGFP^ cells were mixed with primary human bone-marrow mesenchymal cells, collagen type I as extracellular matrix component and with 1 nM of the respective target compound and grafted on the chorioallantoic membrane of chicken embryos (n=3). After five days tumors can be visualized by eGFP expression. In grafts, all drugs showed significant lower fluorescence than the control. Bars indicate 500 μm. (B) Single MM xenografts were excised and homogenized in lysis buffer and subsequently measured using a GFP ELISA. GFP concentrations of single tumors were calculated (n=6, Mean ± SEM). Stars indicate p values <0.05. con.: control.

EGFP-transgenic myeloma cells (OPM-2^eGFP^ and RPMI-8226^eGFP^) together with *ex vivo*-derived human bone-marrow mesenchymal cells were embedded in collagen type I as extracellular matrix component. Test substances were applied topically at 1 nM concentration. These “onplants” were grafted on the chorioallantoic membrane of chicken embryos (using six replicates). After five days, MM xenografts formed tumors that could be visualized by eGFP expression (Figure 2A). Compared to controls (0.1% DMSO), all analyzed target compounds inhibited growth of MM cells in xenografts resulting in less green-fluorescent MM tumor cell mass (Figure 2A). Single MM xenografts (n=6/group) were excised, homogenized and thereafter measured by GFP ELISA. All tested compounds significantly reduced myeloma cell mass in a manner similar to that observed for bortezomib (Figure 2B). Again Zalypsis and PM00113 were the most potent inhibitors of myeloma growth *in vivo*, exceeding even the anti-myeloma activity of bortezomib.

In addition, we observed that revascularization of xenografts and microvessel sprouting adjacent to grafts were significantly reduced by marine-derived compounds (Figure 2A).

### Anti-angiogenic activities of target compounds *in vivo* in the gelatin sponge assay

**Figure 3:**
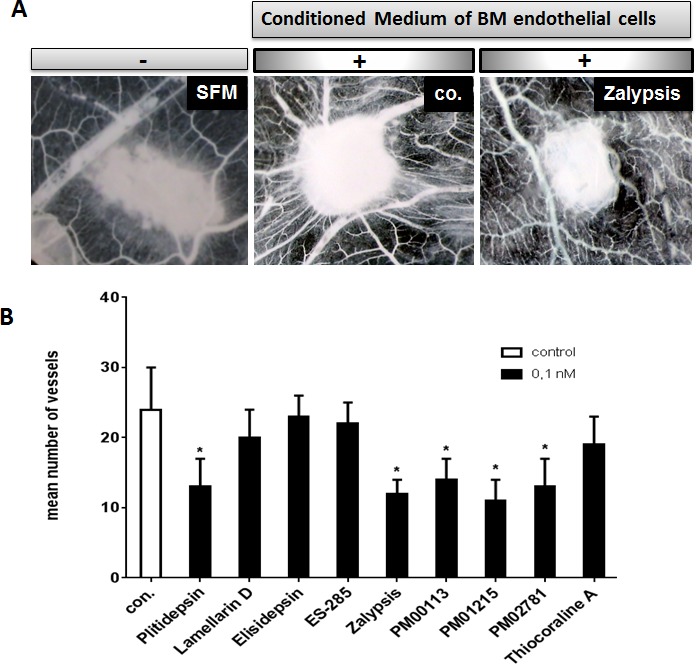
Anti-angiogenic activities of marine-derived compounds in the gelatin sponge assay (A) CAMs were incubated with gelatin sponges loaded with SFM (left) and with conditioned medium of multiple myeloma-derived endothelial cells either alone (co.) or supplemented with 0.1 nM of marine-derived compounds (drug). Note the decreased number of blood vessels. Images were acquired with a stereomicroscope (magnification, x 50). (B) A set of marine-derived compounds tested at 0.1 nM demonstrated anti-angiogenic activity in response to conditioned media of endothelial cells deriving from patients suffering from multiple myeloma (p <0.05). The mean number of vessels in the control was 24±6. Stars indicate p values <0.05.

To investigate whether marine-derived compounds could also inhibit myeloma-related angiogenesis, we tested all compounds in the *in vivo* CAM assay. Sponges soaked with conditioned medium of bone marrow-derived endothelial cells from patients with MM were grafted with compounds S1-S9 on chicken chorioallantoic membranes (CAMs). CAMs implanted with a gelatin sponge together with conditioned medium were surrounded by a rim of newly formed capillaries converging radially toward the sponge in a “spoked-wheel” pattern (mean number of vessels: 24±6; Figure 3A; with serum-free medium: mean number of vessels: 9±2). Marine-derived compounds applied at low nM concentrations markedly inhibited the formation of capillaries (Figure 3A). Addition of compounds at even a concentration of 0.1 nM resulted in a significant inhibition of MM endothelial cell-induced angiogenesis for Plitidepsin, Zalypsis, PM00113, PM01215 and PM02781 (Figure 3B).

## DISCUSSION

Recently, progression-free and overall survival rates in MM patients have been substantially improved by novel classes of therapeutics such as proteosome inhibitors and immunomodulatory drugs (IMIDs) [4, 5]. Nevertheless, virtually all patients relapse and ultimately become resistant to therapy. Thus, scanning for new anti-myeloma drugs and development of combinatorial therapeutic strategies are essential to further improve the outcome of patients with MM. A rapidly growing field of interest is the development of novel drugs that directly target myeloma cells and/or modify the respective microenvironment of MM cells in the bone marrow.

This study evaluated new marine-derived compounds for their anti-myeloma activity *in vitro* and *in vivo* including direct and indirect effects on myeloma cells. A subset of a total of 9 compounds tested (Plitidepsin [S1], Zalypsis [S5], PM00113 [S6] and Thiocoraline A [S9]) demonstrated significant proapoptotic activity in primary human myeloma cells even at 10 nM concentrations, comparable to bortezomib as a standard. *In vitro* primary human myeloma cells were more sensitive to the substances than were established myeloma cell lines. The same set of marine-derived drugs proved to be growth-inhibitory in human MM xenografts *in vitro* and *in vivo.* Plitidepsin blocks cell proliferation by arresting the cells in transition from G0/G1 to S phase and triggers apoptosis by inducing cytochrome c release and activating caspase 9 and 3 [10]. In addition, it also exerts anti-angiogenic activity by reducing the secretion of VEGF and the expression of VEGF receptor-1 from MOLT-4 human leukemia cells *in vitro* [11]. Currently, this compound is undergoing clinical testing in a phase III trial for the treatment of relapsed/refractory MM (registered with www.clinicaltrials.gov, number NCT01102426). Our 3D *in vitro* co-culture data and *in vivo* xenograft data strongly support the development of Plitidepsin as an anti-myeloma drug.

Zalypsis is able to induce cell cycle arrest and apoptosis by provoking double-strand DNA breaks [12]. The strong anti-myeloma effect of this agent has already been described in MM cell lines resistant to standard anti-myeloma treatment [12]. This drug has already been suggested by Ocio and coworkers as one of the most powerful agents that have been evaluated against multiple myeloma [12]. Their findings were confirmed by our MM model systems and extended by analysis of MM primary cells. PM00113, an analog of Zalypsis, is considered to have a similar mode of action, i.e. DNA-binding and inducing double-strand breaks. This new marine-derived compound showed potent anti-myeloma effects at low nM concentrations *in vitro* on MM cell lines (10 nM) as well as on primary human myeloma cells (10 nM) and on GFP-transgenic myeloma-xenografts *in vivo* (1 nM). Thus, further development of the Zalypsis analog for MM is warranted.

Thiocoraline A is a marine-derived cytotoxic antibiotic. This compound causes an arrest in G1 phase and decreases the rate of S phase progression towards G2/M phases due to DNA polymerase-α inhibition [13]. In our MM cell lines and primary MM cells, Thiocoraline A showed potent inhibition of myeloma growth in a dose-dependent manner.

For the purpose of future clinical drug development we conducted a more detailed analysis of the four marine agents effective against primary myeloma cells. Primary bone marrow-derived myeloma cells from MM patients even with high-risk cytogenetic features and progressive disease were remarkably sensitive to marine-derived compounds. As an example, although patient #3 was refractory to treatment with dexamethasone and bortezomib, myeloma cells from his bone-marrow were resistant to bortezomib *in vitro* but were readily killed by compounds Plitidepsin, Zalypsis and PM00113 even at low nanomolar concentrations (10 nM), suggesting the lack of cross-resistance between bortezomib and these marine-derived substances.

Zalypsis and PM00113 were the most potent inhibitors of myeloma growth, exceeding the anti-myeloma activity of bortezomib. Since our experiments showed no cross-resistance to bortezomib, synergies of these marine-derived compounds with established anti-MM drugs including bortezomib and IMIDs can be expected and should be evaluated.

Apart from directly affecting myeloma cells, some of the compounds (Plitidepsin, Zalypsis, PM00113) can also modify the myeloma-associated microenvironment, e.g. via the inhibition of myeloma cell-induced formation of new blood vessels. *In vivo* experiments using the CAM assay revealed significant suppression of myeloma-induced neoangiogenesis close to xenografts and diminished revascularization of xenografts following exposure to marine agents, even at low concentrations (0.1 nM).

Finally, to screen for drug toxicity *in vitro* we analyzed the proapoptotic activity of marine-derived compounds on normal blood mononuclear cells using flow cytometry. All four compounds with significant anti-myeloma activity *in vivo* also induced apoptosis of PBMNC and bone marrow-mesenchymal cells *in vitro,* but only at high drug concentrations (100 nM). We thus conclude that the proapoptotic effects of selected marine compounds are dose-dependent and limited to neoplastic cells when these drugs are applied at drug concentrations of 10 nM or less.

In conclusion, we identified a set of new marine compounds with strong anti-myeloma activity *in vitro* and *in vivo,* representing valid candidates for further preclinical and clinical development in myeloma. Our data indicate no cross-resistance of these drugs with bortezomib, suggesting that they could be used in combination with established anti-myeloma drugs.

## MATERIALS AND METHODS

### Ethics statement

Investigations have been conducted in accordance with the ethical standards and according to the Declaration of Helsinki and according to national and international guidelines and have been approved by the authors' institutional review board.

### Reagents

All marine-derived compounds were synthesized by PharmaMar (Colmenar Viejo, Madrid, Spain), stocked in DMSO at a concentration of 1 [mM] and stored at −20° C. Bortezomib was purchased from Selleckchem and dissolved in DMSO (SIGMA Biochemicals) as a stock solution of 250 mM. Tunicamycin was purchased from Sigma. All compounds were freshly diluted in culture medium immediately before use.

Plitidepsin (Aplidin) [S1] is a depsipeptide discovered in the tunicate *Aplidium albicans* [14]. Lamellarin D [S2] was initially isolated from a marine mollusk of the *Lamellaria sp.* [15] and subsequently identified from ascidians [16]. The synthetic antitumor agent Elisidepsin [S3] is structurally related to Kahalalide F*,* originally isolated from the Hawaiian marine mollusk *Elysia rufescens* [17]. The compound ES-285 [S4] was discovered in the sea mollusk *Spisula polynyma* [17]. Zalypsis [S5] is a synthetic alkaloid, related to the marine natural compound Jorumycin and the family of Renieramycins originally obtained from pacific mollusks and sponges [18, 19]. Zalypsis has been shown to exert potent antimyeloma activity by inducing DNA double-strand breaks [12]. PM00113 [S6], an analog of Zalypsis, is suspected to have a mode of action similar to that of tetrahydroisoquinoline alkaloids. PM01215 [S7] and PM02781 [S8] are two novel synthetic analogs of Plitidepsin. Thiocoraline A [S9]*,* a cytotoxic antibiotic, was originally isolated from the actinomycete *Micromonospora marina*, found in southeast Africa [20]. Chemical structures of all compounds are shown in Supplementary Figure 4.

### Cell culture

The MM cell lines OPM-2 (ACC 50), U266 (ACC 9), NCI-H929 (ACC 163), and RPMI-8226 (ACC 402) were purchased 2012 directly from DSMZ (Germany), authenticated by us by flow cytometry (CD138+/CD38+) as well as by STR-profiling (data shown in Supplementary Table 2) and cultivated in RPMI1640 medium (Sigma Aldrich), supplemented with 10% bovine calf serum (Hyclone) and 100 IU/mL penicillin, 100 μg/mL streptomycin and 2 mM glutamine (all PAA Laboratories GmbH) in the presence of 5% CO_2_ at 37°C. OPM-2 and RPMI-8226 cells were lentivirally transfected to express eGFP and propagated in the presence of blasticidin (2.5μg/mL, Invitrogen) before usage for *in vivo* experiments.

Peripheral blood mononuclear cells (PBMNC) were separated from heparinized blood from five healthy volunteers by Lymphoprep (PAA Laboratories GmbH) gradient centrifugation, washed three times in Dulbecco's Phosphate-Buffered Saline (DPBS; Bio Whittaker), counted and resuspended in the above mentioned culture medium.

Human mesenchymal stem cells from bone marrow from three healthy donors were purchased from PromoCell. Cells were cultivated in MM cell line culture medium on uncoated plastic material.

Primary myeloma cells were obtained from heparinized bone marrow aspirates from patients with active MM (n=10; for details see Supplementary Table 1) following approval by the local ethics committee and the patients' written informed consent. Aspirates were cultivated in MM cell line culture medium in uncoated Falcon plastic flasks.

### Detection of cell viability by flow cytometry

PBMNC and MM cells (cell lines and CD38^high^ /CD45^low^ primary myeloma cells) were seeded into nutrient medium in 96-well plates (100,000 cells/well). Marine-derived drugs and bortezomib as reference substance were applied at concentrations of 0.1-100 nM. Cells were exposed to the various drugs for 24 hours in an atmosphere of 5% CO_2_ at 37°C. All experiments were done in triplicate. Cell immunostaining was performed with antibodies directed against human CD45 (label-FITC) and CD38 (label-PE) purchased from Miltenyi BD Biosciences.

Cell viability was determined by annexin V APC (Mab Tag) /7-AAD (7-amino-actinomycin D; Beckman Coulter) staining in a binding buffer consisting of 40 mM N-2-hydroxyethylpiperazine-N-2-ethanesulfonic acid, pH 7.4, 560 mM NaCl, and 10 mM CaCl_2_. Stained cells were measured on the flow cytometer (FACSCanto^TM^ II, BD Biosciences) and analyzed with the BD FACS DIVA software. Results were expressed as percentage of viable cells, i.e. % of living cells ± standard error of the mean (SEM).

### Generation of eGFP-encoding lentiviruses

The GFPmut1 chromophore is available in a codon-optimized version (eGFP) in the commercial vector pCMS eGFP (Invitrogen). The eGFP open reading frame with 3′ UTR and polyadenylation signal was cloned by amplifying the fragment by means of KOD polymerase (Novagen) and specific forward (eGFP-for: 5-tgcaaaaagcttgccacaac) and reverse primers (5 eGFP-rev: 5-ATTGTCTCATGAGCGGATAC).

The resulting PCR product was ligated into the pENTR11 Gateway vector (Invitrogen, Vienna, Austria), predigested with Xmn-I and Eco RV (NEB) and polished with Klenow. All amplified and purified pENTR11 vectors were control-sequenced for correct orientation and exclusion of incorporated mutations. pENTR 11 vectors were site-specifically recombined with the pLenti6-V5 DEST vector (Invitrogen) using the Gateway LR Clonase II Pus Enzyme Mix (Invitrogen). The resulting pLenti6 DEST vector with eGFP open-reading frame was transformed and propagated in One-Shot Stabl3 bacteria (Invitrogen). Lentiviruses were produced in HEK293FT cells by transfecting cells with the pDEST6 vectors and helper plasmid mix (ViraPower, lentiviral support kit) using Lipofectamine 2000 (both from Invitrogen). Lentiviral titers were determined by real time PCR and quantification of woodchuck hepatitis virus posttranscriptional response element expression (WPRE-for: 5-actgacaattccgtggtgtt; WPRE-rev: 5-agatccgactcgtctgagg). Cell lines were lentivirally transfected (pDEST6 eGFP) and stable cell lines selected with 2 μg/mL blasticidin (Invitrogen). Cell lines expressing the green reporter eGFP after two weeks of selection were named OPM-2^eGFP^ and RPMI-8226^eGFP^.

### 3D myeloma spheroid model

Transgenic MM cell lines (OPM-2^eGFP^ and RPMI-8226^eGFP^; 250,000 per spheroid) were mixed into the collagen together with human mesenchymal stromal cells (50,000 cells/spheroid) or with the respective concentrations of compounds (1-100 nM). Aliquots of the collagen/cell mixture (30 μl) were distributed over a paraffin film in a 12-well plate and allowed to polymerize for 30 min at 37°C, generating so-called collagen spheroids. Thereafter, spheroids were overlaid with culture medium containing the respective concentrations of compounds (1-100 nM) and cultivated for three days. After 72 hours of incubation at 37%, the spheroids were documented with the Olympus SZX10 stereomicroscope (Olympus) equipped with an Olympus DFPL 2X-4 objective lens (numerical aperture 0.2) connected to a digital camera (Olympus E410) and flexible cold light (KL200; Olympus).

### Multiple myeloma xenograft model using the CAM assay

The chick chorioallantoic membrane (CAM) assay is an established *in vivo* model that enables the investigation of tumor angiogenesis, metastasis and invasion without the need to sacrifice mature animals [21, 22]. In brief, fertilized white Leghorn chicken eggs (SPF eggs; each group n=5) were purchased from Charles River and cultivated in an egg incubator at 37°C and 70% humidity (Compact S84) for three days. Thereafter, embryos were transferred to a plastic dish and incubated “*ex ovo*” for further four days, so that the CAM was able to develop. OPM-2^eGFP^ and RPMI-8226^eGFP^ myeloma cells (2.5 × 10^5^) were mixed with rat-tail collagen and human bone-marrow mesenchymal stromal cells (0.5 × 10^5^) and the 1 nM of the respective compounds. Collagen drops were placed on parafilm for 30 min to allow polymerization of the extracellular matrix at 37°C. Then onplants were transferred to the CAM of 7-day-old chicken embryos. After five days of *in vivo* growth, onplants were documented with the Olympus SZX10 stereomicroscope (Olympus) equipped with an Olympus DFPL 2X-4 objective lens (numerical aperture 0.2) connected to a digital camera (Olympus E410) and flexible cold light (KL200; Olympus).

### Quantification of eGFP protein by ELISA

MM spheroids or excised xenografts were transferred to 0.5 ml RIPA Buffer (Sigma Aldrich) and homogenized with an Ultra Turrax Homogenizer three times for 5 sec each on ice. Thereafter, homogenate underwent three freezing/thawing cycles in liquid nitrogen and a 37°C water bath. After centrifugation supernatants were diluted in assay buffer. GFP levels were measured with GFP ELISA Kit (Cell Biolabs) using biotinylated anti-GFP antibodies according to the manufacturer's protocol.

### The gelatin sponge-chorioallantoic membrane assay

Fertilized white Leghorn chicken eggs were incubated at 37°C and constant humidity [23]. On day 3, the shell was opened and 2 mL to 3 mL of albumen was removed to detach the chorioallantoic membrane (CAM). On day 8, the CAMs were implanted with 1-mm^3^ sterilized gelatin sponges (Gelfoam Upjohn) loaded with serum-free medium (SFM) alone (negative control) or CM of multiple myeloma endothelial cells (isolated accordingly Vacca et al. [24] alone (positive control) or with 0.1 nM of each compound tested. The angiogenic response was evaluated on day 12 as the number of vessels converging toward the sponge at a magnification of 50X and photographed *in ovo* (Olympus stereomicroscope).

### Statistical analysis

Statistical analyses were performed with the GraphPad Prism^TM^ software for Windows. All tests of statistical significance were two-sided. Student's t-test, two-way ANOVA and Mann-Whitney U tests were used to study differences between the two groups.

## SUPPLEMENTARY MATERIAL FIGURES AND TABLES


