# Supplementary material for: Marine compounds inhibit growth of multiple myeloma in vitro and in vivo
Source: Oncotarget. 2015 Jan 31;6(10):8200–9. doi: 10.18632/oncotarget.3362 (PMC4480745; doi:10.18632/oncotarget.3362)
Supplement: Supplementary file 1 [file oncotarget-06-8200-s001.pdf]

Marine compounds inhibit growth of multiple myeloma *in vitro* and *in vivo*

Supplementary Material

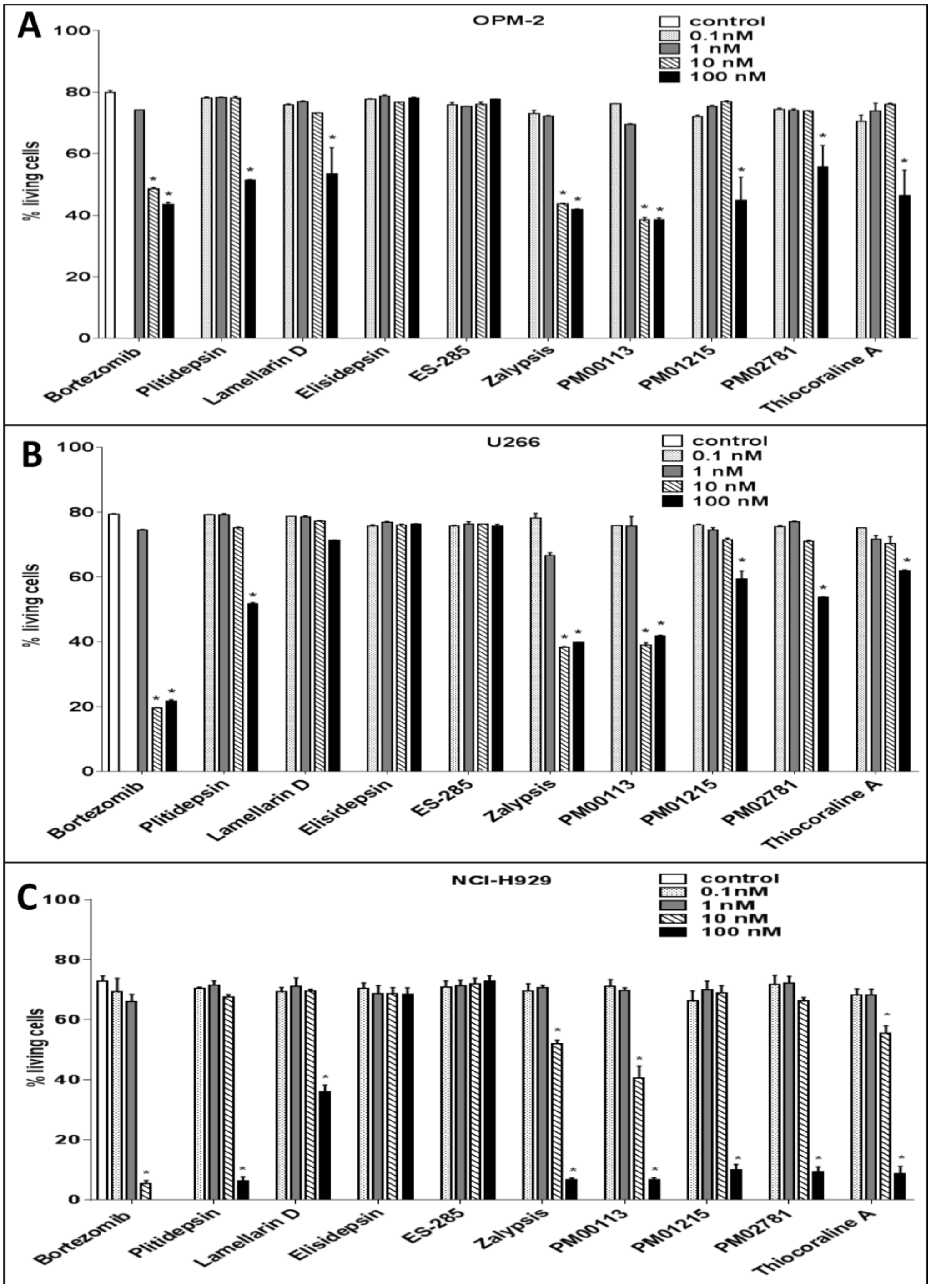

Supplementary Figure 1: Induction of apoptosis by marine-derived compounds in MM cell lines. MM cell lines were incubated with increasing concentrations of drugs (S1-S9) for 24h and then analyzed for viable cells by flow cytometry. Annexin-V^{neg} and 7AAD^{neg} cells were defined as viable. Experimental data from multiple myeloma cell lines OPM-2 (A) U266 (B) and NCI-H929 (C). Mean of triplicates ± SEM; stars indicate p values <0.05.

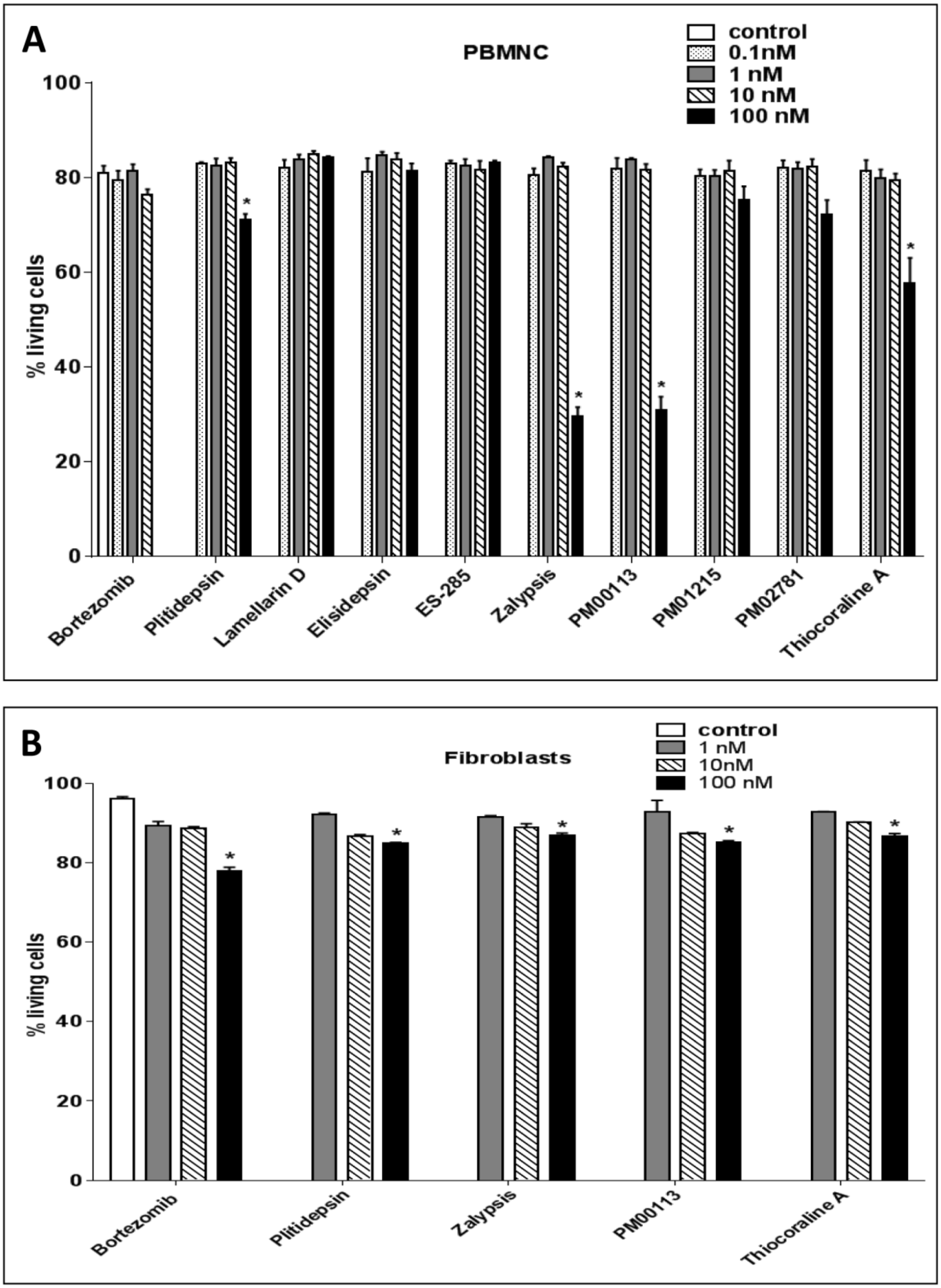

Supplementary Figure 2: Induction of apoptosis by marine-derived compounds in human mesenchymal cells from bone marrow and peripheral blood mononuclear cells. Human peripheral blood mononuclear cells (A) and primary mesenchymal cells from bone marrow (B) were incubated for 24h with increasing concentrations of target compounds and then analyzed for viable cells by flow cytometry. Annexin-V^{neg} and $7AAD^{neg}$ cells were defined as viable. Mean of triplicate results ± SEM; stars indicate p values <0.05.

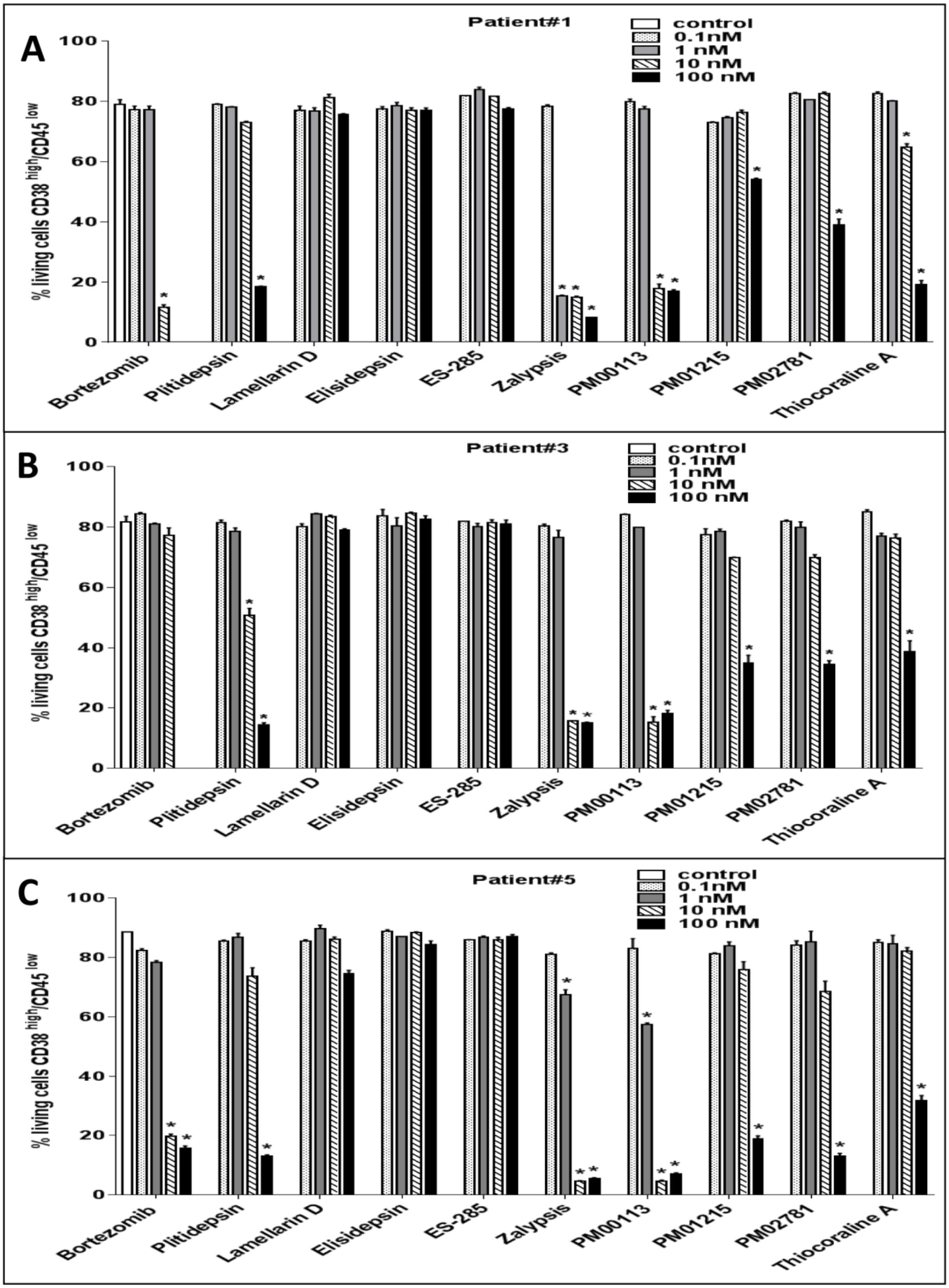

Supplementary Figure 3: Induction of apoptosis by marine-derived compounds in primary bone marrow aspirates. Primary MM cells from bone marrow aspirates were incubated with increasing concentrations of drugs (S1-S9) for 24h and then analyzed for viable cells by flow cytometry. Annexin-V^{neg} and $7AAD^{neg}$ cells were defined as viable. Experimental data from multiple myeloma patient #1 (A), patient #3 (B) and patient #5 (C). Mean of triplicates ± SEM; stars indicate p values <0.05.

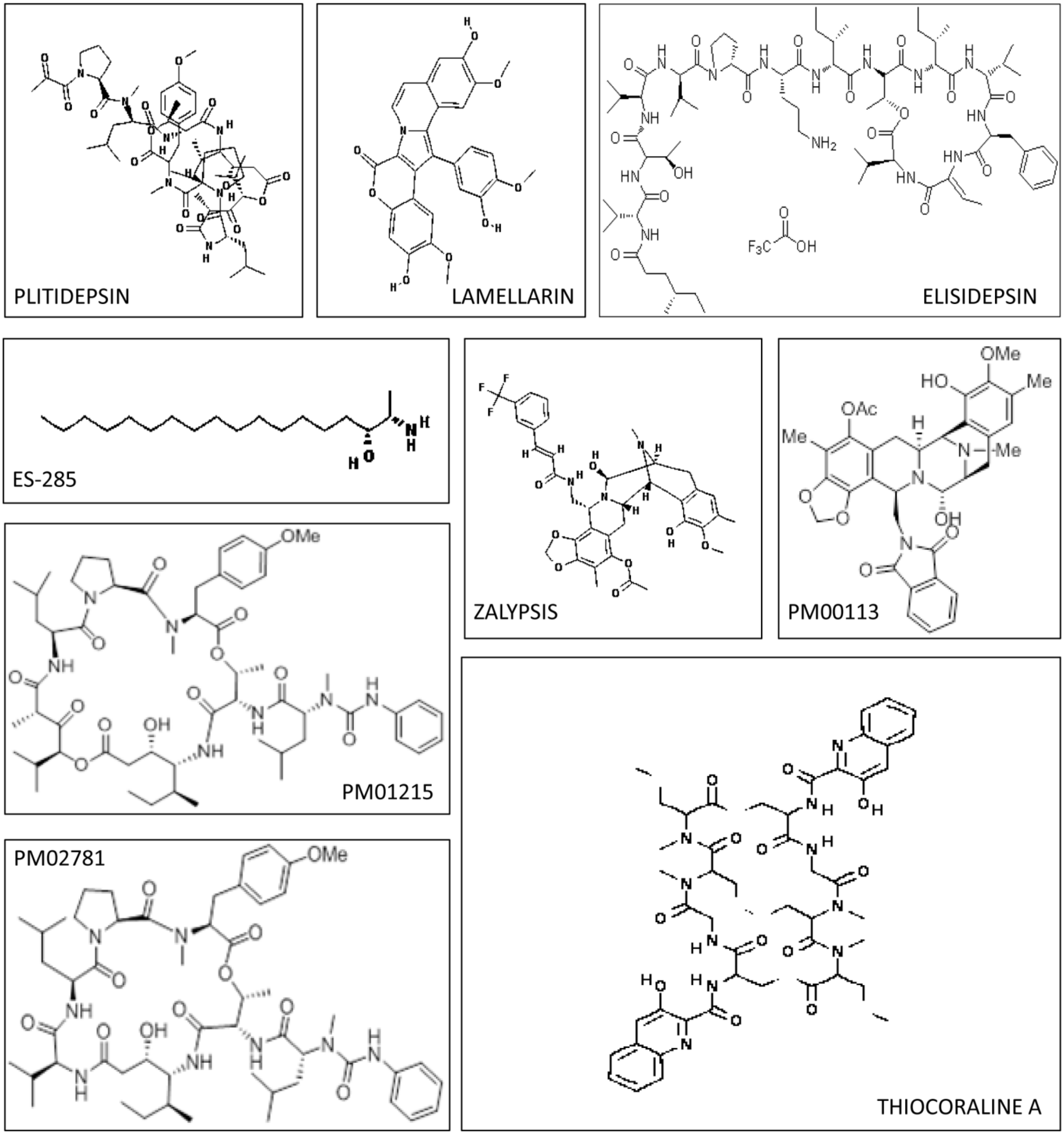

Supplementary Figure 4: Chemical structures of the tested marine-derived compounds.

Supplementary Table 1: Patient and disease characteristics

patient	sex	age	stage (ISS)	isotype	karyotype	fish	history	treatment
MM#1	M	65	I	IgG Kappa	*46XY t(4;14)*	*del 13q14*	MM; newly diagnosed	**BTZ/thalidomide/dexamethasone (VTD) 1st line**
MM#2	M	59	II	IgG Kappa	*46XY*	*del 11q22*	PD	BTZ/cyclophosphamide/dexamethasone (VCD) 1st line<br>**BTZ/lenalidomide/dexamethasone (VRD) 2nd line**
MM#3	M	82	II	IgG Kappa	*46XY t(11;14)*	*IGH Rearrange-ment*	MGUS<br>PD	**BTZ/dexamethasone (VD) 1st line**
MM#4	F	67	II	IgG Lambda	*46XX*	*del 11q22*	relapsed disease	BTZ/thalidomide/dexamethasone (VTD); ASCT 1st line<br>**BTZ/thalidomide/dexamethasone (VTD) 2nd line**
MM#5	F	64	II	IgG Kappa	*46XX t(4;14)*	*amp 1q21*<br>*del 13q14*<br>*del 17p*	PD<br>PCL	BTZ/thalidomide/dexamethasone (VTD); ASCT 1st line<br>lenalidomide maintenance therapy 2nd line<br>**pixantrone/dexamethasone/pomalidomide 3rd line**
MM#6	F	75	I	Light chain kappa	*46XX*	*del 13q14*<br>*amp 1q21*	PD	BTZ monotherapy 1st line<br>lenalidomide/dexamethasone 2nd line<br>bendamustine/BTZ/dexamethasone 3rd line<br>pomalidomide 4th line<br>**oral cyclophosphamide 5th line**
MM#7	M	81	II	IgG Kappa	*46XY t(4;14)*	*amp 1q21*<br>*del 11q22*<br>*del 13q14*	PD<br>PCL	**carfilzomib/melphalan/prednisone** 1st line
MM#8	F	66	III	Light chain kappa	*46XX*	_	PCL; newly diagnosed	**BTZ/lenalidomide/dexamethasone (VRD) 1st line**
MM#9	M	71	I	IgA Kappa	*46XY*	*amp 1q21*<br>*del 11q22*<br>*del 17p*	PD	BTZ monotherapy; ASCT 1st line<br>lenalidomide monotherapy 2nd line<br>BTZ monotherapy 3rd line<br>**pomalidomide/dexamethasone 4th line**
MM#10	F	61	III	Light chain kappa	*46XX t(11;14)*	*del 13q14*	PD	BTZ/dexamethasone (VD) 1st line<br>BTZ/thalidomide/dexamethasone (VTD) 1st line<br>lenalidomide monotherapy 2nd line<br>lenalidomide/bendamustine 3rd line<br>ASCT 4th line<br>lenalidomide/dexamethasone (RD) 5th line<br>BTZ/doxorubicine 6th line<br>oral cyclophosphamide/dexamethasone 7th line<br>**pomalidomide/dexamethasone 8th line**

In patient #8, cytogenetics is not available (-). PD: progressive disease; MGUS: monoclonal gammopathy of undetermined significance; PCL: plasma cell leukemia; BTZ: bortezomib; ASCT: autologous stem cell transplantation.

Supplementary Table 2: Cell line characterization by STR-profiling (OPM-2, NCI-H929, U266, RPMI-8226)

Cell name	Locus names								
	D5S818	D13S317	D7S820	D16S539	VWA	TH01	AM	TPOX	CSF1PO
**Used cells**	13,13	11,11	12,12	9,13	14,17	6,7	X	8,8	12,13
**OPM-2**	13,13	11,11	12,12	9,13	14,17	6,7	X,X	8,8	12,13

Cell name	Locus names								
	D5S818	D13S317	D7S820	D16S539	VWA	TH01	AM	TPOX	CSF1PO
**Used cells**	11,12	12	10,12	9,13	14,15	9,3	X	8,11	11
**NCI-H929**	11,12	12	10,12	9,13	14,15	9,3	X,X	8,11	11

Cell name	Locus names								
	D5S818	D13S317	D7S820	D16S539	VWA	TH01	AM	TPOX	CSF1PO
**Used cells**	11,12	12	11,12	10	17	5,7	X,Y	8	12,13
**U266**	11,12	12	11,12	10	17	5,7	X,Y	8	12,13

Cell name	Locus names								
	D5S818	D13S317	D7S820	D16S539	VWA	TH01	AM	TPOX	CSF1PO
**Used cells**	11,13	11,11	9,10	9,9	16,16	8,8	X,Y	8,11	12,12
**RPMI-8226**	11,13	11,11	9,10	9,9	16,16	8,8	X,Y	8,11	12,12

OPM-2, NCI-H929, U266, RPMI-8226 cells were identified by Short Tandem Repeat analysis (STR analysis, Identifiler® Life Technologies). STR analysis revealed that STR patterns of OPM-2, NCI-H929, U266, RPMI-8226 cells were identical with the respective reference foci provided by DSMZ (German Collection of Microorganisms and Cell Cultures).
